# Homocysteine and lipid profile in children with Juvenile Idiopathic Arthritis

**DOI:** 10.1186/1546-0096-5-2

**Published:** 2007-04-02

**Authors:** Marcela Gonçalves, Vânia D'Almeida, Elvira M Guerra-Shinohara, Luciano C Galdieri, Claudio A Len, Maria Odete E Hilário

**Affiliations:** 1 Department of Pediatrics, Universidade Federal de São Paulo (Unifesp), São Paulo, Brazil; 2 Department of Clinical Chemistry and Toxicology, Faculty of Pharmaceutical Science, Universidade de São Paulo (USP), São Paulo, Brazil

## Abstract

**Background:**

An increased concentration of plasma homocysteine (Hcy) has toxic effects on vascular endothelium. This seems to be a risk factor of cardiovascular disease, premature stroke and venous thrombosis. The risk is higher in coincidence with other factors like chronic diseases and familiar hypercholesterolemia. The aim of our study was to evaluate plasma Hcy concentration in patients with juvenile idiopathic arthritis (JIA) and its correlation with methotrexate (MTX) therapy, serum folate and B12 vitamin, and hyperlipidemia.

**Methods:**

Fifty-one patients (37 females; mean age 11.3 years, range 2.3–17 years) with JIA and 52 healthy controls (42 females; mean age 12.5 years; range 3–18 years) were included in the study. Thirty-two patients were using weekly MTX (mean doses: 0.1–1 mg/kg). For statistical analysis both JIA and control groups were distributed in three subgroups according to age (3 – 7, 8 – 12 and 13 – 18 years). The laboratory investigation included measurement of erythrocyte sedimentation rate (ESR), C-reactive protein (CRP), plasma Hcy, serum folate, vitamin B12, triglycerides, total cholesterol, high-density lipoprotein (HDL), low-density lipoprotein (LDL) and very low-density lipoprotein (VLDL). For data analysis, we considered raised Hcy values ≥ 12.56 μmol/L, which corresponds to the 90^th ^percentile observed in the control group.

**Results:**

The mean plasma Hcy concentration was 9.3 ± 3.16 μmol/L in JIA patients and 8.9 ± 2.42 μmol/L in healthy controls (p = 0.615). Higher concentration of Hcy was observed in the subgroup of 13 – 18 years (patients and controls, p < 0.001). We did not find correlation between MTX use and plasma Hcy concentration. With regard to vitamin B12 concentration, we detected normal values in both patients and controls while serum folate concentration was higher in patients (p < 0.001). With regard to the lipidogram, lower concentration of HDL was found in patients (p = 0.007) and higher levels of VLDL (p = 0.014) and triglycerides (p = 0.001) were observed in controls. We did not observe correlation among plasma Hcy concentration, clinical findings, ESR and CRP.

**Conclusion:**

JIA patients do not present significant increased concentration of Hcy despite the use of MTX, probably due to the folate supplementation. The mild abnormalities in the lipidogram may reflect a current concern with diet and health.

## Background

Homocysteine (Hcy) is a sulphydryl amino acid derived from the essential amino acid methionine during its conversion to cysteine [1]. Its main metabolic pathways require folic acid and vitamins B6 and B12. Its concentrations depend on age, gender and ethnic background [2].

Hcy has many functions: threshold of the auto-oxidation of low-density lipoprotein (LDL) [3], promote vascular smooth-muscle cell proliferation, platelet activation and endothelium dysfunction [4].

Both in children and in adults increased concentration of plasma Hcy has direct and indirect toxic effects on the vascular endothelium and seems to be one more risk factor of cardiovascular disease, premature stroke and venous thrombosis [5], especially when associated with other risk factors like familiar hypercholesterolemia.

Juvenile idiopathic arthritis (JIA) is a chronic inflammatory disease in which clinical manifestations may persist for decades in some patients despite adequate treatment [6]. There are two studies about the level of Hcy in children with JIA and the results have been conflicting [7,8]. Huemer [7] et al evaluated Hcy levels in 17 patients with JIA using methotrexate (MTX) with or without folic acid supplementation and in controls matched for age and gender. The average Hcy concentration in 17 patients was significantly higher than in the controls. However, a relation between the Hcy concentration and treatment with MTX or folic acid was not found. The same authors [8] further evaluated 35 JIA patients and healthy controls with the purpose of determining the Hcy concentration and genetic polymorphism capable of increasing the Hcy concentration in this population. In this study the Hcy concentration was lower in the patients than in the controls.

The paucity of studies about this subject and the conflicting results published to the present date stimulated us to evaluate this metabolite in children and adolescents with JIA.

## Methods

### Subjects

A cross-sectional study involving 51 children and adolescents with JIA (37 girls, current average-age 11.3 years) selected consecutively from our outpatient Pediatric Rheumatology Unit was performed. All of them were diagnosed according to the International League of Associations for Rheumatology (ILAR) [9]. Fifty-two "healthy" children and adolescents (42 girls, current mean-age 12.5 years) selected from a public school in the same area were included as controls. None of the controls presented a family history of autoimmune disease. Both patients and controls denied cigarette or alcohol use.

### Disease activity

All patients were clinically evaluated by a board certified pediatric rheumatologist. We defined active JIA when the patient presented one or more joints with arthritis at the time of the study. We also measured the ESR and CRP of all patients.

### Laboratory study

Patients and controls samples were identified by an unknown code by the laboratory staff. Thirty milliliters of blood were collected from fasting participants and centrifuged at 3000 rpm for 5 minutes. The plasma was separated in aliquots and immediately frozen and stored at -80°C. The time between the beginning and the end of this process did not exceed one hour.

One portion of each blood sample was used to determine the total Hcy concentration, the concentration of vitamin B12 and serum folate. The leftover blood sample was used to determine the total cholesterol level (TC) and its fractions, and triglycerides (TG).

Total plasma Hcy concentration was measured according the method proposed by Pfeiffer et al, which uses the High Performance Liquid Chromatography (HPLC) with fluorimetric detection and isocratic elution [10]. Increased Hcy concentration was defined as plasma Hcy concentration equal to or higher than 12.56 μmol/L (90^th ^percentile of the control group).

Vitamin B12 concentration was performed by an enzyme immunoassay method with micro particles (Imx B12 ^® ^of Abbot Laboratories). Serum folate concentrations were measured by ionic capture (Imx Folate ^® ^of Abbot Laboratories).

TC, high-density lipoprotein (HDL), LDL, very low-density lipoprotein (VLDL), serum creatinine as well as TG were measured by the enzymatic colorimetric method.

### Statistical Analysis

For statistical analysis both JIA and control groups were distributed into 3 subgroups according to age (3 – 7 year-old, 8 – 12 year-old and 13 – 18 year old).

To establish a reference value (cutting point) in order to make comparisons between the groups of patients and controls, a receiver operating characteristics (ROC) curve analysis was conducted and after that, the categorization by the 90^th ^percentile of the control group.

Some variables like Hcy concentration, TC and its fractions, TG, vitamin B12, serum folate concentrations and serum creatinine were transformed into logarithm (Log_10_) due to the variability and asymmetry of values, in order to obtain a normal probability distribution.

For the comparison of categorical variables of Hcy, the chi-square test was used, and when necessary (expected values lower than five), the Fisher's exact test was used. The Student's t-test was used for the comparison of continuous variables. The relation between Hcy concentration and other continuous variables was analyzed by Spearman's correlation coefficient.

To evaluate the relation between high and normal Hcy concentration groups of normal and high values and the other variables in each group, the logistic regression analysis with logic model was used to estimate odds ratio. Univariate and multivariate analysis were conducted using the stepwise variables selection criteria.

The level of significance adopted for the statistical tests was 5%, that is, p < 0.05.

The study protocol was approved by the Ethics Committee of the Universidade Federal de São Paulo. A consent form was obtained for each patient and control either from a parent, guardian or responsible adult before inclusion in the study.

## Results

The demographic and clinical characteristics of the patients are presented in table 1. We observed a predominance of the female gender (72.6%) and oligoarticular onset type (43.1%); 52.9% of the patients had active disease and 62.7% were taking MTX.

**Table 1:** Demographic and clinical characteristics of patients with juvenile idiopathic arthritis (N = 51) and controls (N = 52).

	Patients		Controls	
Epidemiological Characteristics	n	(%)	n	(%)
Gender				
Female	37	(72.6)	42	(80.1)
Male	14	(27.4)	10	(19.9)
Origin				
Caucasian	38	(74.5)	27	(51.9)
Non Caucasian	13	(25,5)	25	(48.1)
Age at disease onset (years)				
Range	1–12		-	
Mean ± SD	5.6 ± 3.2		-	
Median	5.8		-	
Current age (years)				
Medium ± DP	11.3 ± 4.2		12.5 ± 3.5	
Median	12.3		13	
Onset type JIA				
Oligoarticular	22 (43.1%)		-	
Polyarticular	17 (33.3%)		-	
Systemic	12 (23.6%)		-	
Course type				
Oligoarticular	29 (56.9%)		-	
Polyarticular	22 (43.1%)		-	
				
Active disease treatment	27 (52.9%)			
Methotrexate	32 (62.7%)			
NSAIDs	26 (51%)			
Chloroquine	10 (13.9%)			
Folic acid	32 (62.7%)			
Systemic steroids	4 (7.8%)			

**JIA**: juvenile idiopathic arthritis, **NSAID**s: nonsteroidal anti-inflammatory drugs

**Figure 1:**
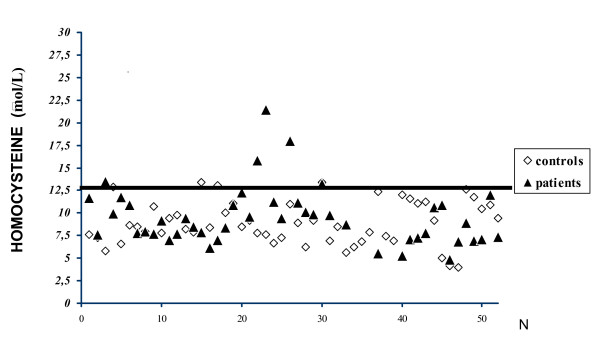
Homocysteine concentration in patients (N = 51) and controls (N = 52) (categorized by p = 90%).

Average serum Hcy concentration was 9.3 ± 3.16 μmol/L in JIA patients and 8.9 ± 2.42 μmol/L in healthy controls (p = 0.615) (Table 2). Only 5 patients (9.8%) and 5 controls (9.6%) presented high levels of Hcy (Figure 1). Considering the age groups, higher average plasma Hcy concentration was observed in patients from 13 to 18 years of age (p < 0.001) (Table 3). No difference in the Hcy concentration was observed between gender in patients (p = 0.293) and controls (p = 0.794).

**Table 2:** Hcy, vitamin B12, folate, cholesterol fractions and triglycerides concentrations in patients (n = 51) and controls (n = 52).

	Hcy	Vitamin B12	Folate	VLDL	HDL	LDL	TG
Patients							
mean (SD)	9.27 (3.16)	552 (315.5)	11.25 (4.72)	15.43 (5.19)	39.0 (7.67)	94.75 (20.41)	71.24 (23.33)
Controls							
mean(SD)	8.92 (2.42)	484.5 (246.6)	6.66 (2.27)	20.12 (11.01)	43.58 (9.21)	89.81 (19.15)	93.98 (41.17)
							
p value	0.615	0.341	< 0.001	0.014	0.007	0.208	0.001

Hcy = homocysteine (μmol/L); VLDL = very low density lipoprotein (mg/dL); HDL = high density lipoprotein (mg/dL); LDL = low density lipoprotein (mg/dL); TG = triglycerides (mg/dL)

**Table 3:** Average serum homocysteine concentration of patients with juvenile idiopathic arthritis (n = 51) and controls (n = 52), according to age group.

	Average serum Hcy (μmol/L)	SD
Patients (n = 51)		
3 – 7 year-old (n = 13)	7.29 *	1.45
8 – 12 year-old (n = 16)	8.62	1.87
13 – 18 year-old (n = 22)	10.93 *	3.80
Controls (n = 52)		
3 – 7 year-old (n = 6)	7.42	3.98
8 – 12 year-old (n = 19)	9.51	4.18
13 – 18 year-old (n = 27)	8.92	5.62

Hcy = homocysteine, SD = standard deviation

* Patient subgroups: 3 – 7 year-old vs. 13 – 18 year-old (p < 0.001)

With regard to the analysis of the Hcy concentration, we did not observe significant differences in relation to the onset and course types of disease and MTX. No correlation among Hcy plasma concentration or clinical and laboratory parameters in JIA patients was observed.

Vitamin B12 concentration was normal in patients and controls while serum folate concentration was higher in patients than in controls (11.25 ng/ml ± 4.72 and 6.66 ng/ml ± 2.27 p < 0.001) (Table 2). None of the patients presented folate levels below the value considered normal.

Regarding the lipidogram, 7 patients presented total cholesterol levels varying between the limitrophe and the increased value (p = 0.398) (Table 2). A lower concentration of HDL was found more frequently in patients than in controls (39.00 mg/dL and 43.58 mg/dL, p = 0.007). Higher levels of VLDL (p = 0.014) and triglycerides (p = 0.001) were observed in controls (71.24 × 93.98 mg/dL) (p = 0.001) (Table 2).

## Discussion

JIA is a chronic inflammatory disease that affects young children and adolescents. In this disease the mortality is low, and many patients have a good quality of life in their adult years. Studies with normal adults and rheumatoid arthritis patients have shown a correlation between plasma Hcy concentration and the presence of cardiovascular and thromboembolic events [11,12]. This data is of great importance given that the abnormalities in the Hcy concentration can be changed during childhood and adolescence, leading to a higher survival rate and to a better quality of life. However, in childhood the relationship among Hcy, vitamin B12 and folate has not been well determined yet.

In this study we observed an elevated concentration of plasma Hcy in some children with JIA and also in some controls. We found a significant difference between two groups of patients: 3 to 7 year-old and 13 to 18 year-old (p < 0.001). Some studies relate age to an increase in Hcy levels, especially in children who are over 10 [13-15]. In the control group we observed a higher value in children between 8 and 12 years of age (p = 0.107). However, we did not observe significant differences between the patients and the controls (average = 9.3 μmol/L × 8.9 μmol/L, respectively, p = 0.615).

In the present study we observed a progressive increase in the average concentration of Hcy in patients with JIA and controls related to age.

There are few articles about the subject. Huemer et al [7] studied 17 patients with JIA, all of them using MTX, and 17 controls matched for gender and age. These authors showed patients presented a higher Hcy concentration when compared with the controls (p < 0.04). In another study 35 children with JIA and 62 controls were evaluated. The authors did not find significant increase of the Hcy concentration in both groups [8].

Hcy serum concentration can be a result of several factors including: genetic aspects [13,16], age [14], lifestyle [17], biochemical alterations as folate deficiency [18], certain medications [19] and some diseases [20].

One of the main determinant factors of the Hcy concentration is folate [18], due to its participation in the remethylation process. Studies with healthy children showed a correlation between low serum folate and cobalamin and elevated Hcy [2,14]. In our subjects, abnormalities in concentration of vitamin B12 were not observed; we detected an increase in folate concentration in patients when compared to controls (p < 0.001), probably related to folic acid supplementation.

It is known that MTX takes part in Hcy metabolism increasing its plasmatic concentration [21]. Van Ede et al evaluated 113 patients with rheumatoid arthritis using MTX and concluded that a small dosage of this drug may increase Hcy concentration [22]. According to our results, 4/5 patients with elevated Hcy concentration were taking MTX.

Corticosteroids, diuretics and immunosuppressive drugs like azathioprine and cyclosporine may interfere with Hcy concentration [23]. In our study, a patient with polyarticular JIA used azathioprine and 3 patients with polyarticular course used cyclosporine. Only one of these showed an elevated Hcy concentration.

Chiang et al [24] reported a positive correlation between ESR and Hcy concentration after the methionine load test. Friso et al [25] did not obtain this positive correlation between Hcy concentration and the CRP in 891 subjects. In our study we did not find significant data which could correlate Hcy concentration with ESR and CRP levels.

Dyslipidemia may be found in some rheumatic diseases especially related to disease activity and corticosteroid use [26]. Its relation with the mechanism of arteriosclerosis and cardiovascular diseases is studied worldwide [27]. Dyslipidemia was also reported in children with JIA and systemic lupus erythematosus (SLE). In a study with 37 children with chronic arthritis and 9 with active disease, the levels of TC and fractions, TG and apoliprotein were measured. This study showed an insignificant diminution of LDL in the patients with active disease when compared to either patients without active disease or to controls. This study also showed low concentrations of apoliprotein A1 in patients with disease activity when compared to patients out of activity and controls [28]. In 1988, Ilowite et al [29] studied children with SLE and observed that despite corticosteroid use, increase of Hcy concentration was associated to disease activity. We did not observe higher levels of TC in our JIA patients when compared to controls. With regard to the HDL cholesterol fraction, our patients showed decreased serum levels. This finding was also observed by Urban et al [30] in their studies involving patients with JIA.

## Conclusion

JIA patients do not present significant increased concentration of Hcy despite the use of MTX, probably due to the folate supplementation. The mild abnormalities in the lipidogram may reflect a current concern with diet and health.

Our data is not conclusive. Multicentric studies involving a higher number of patients will help to indicate with more accuracy the significance of Hcy in patients with JIA.

## Abbreviations

Hcy = homocysteine

LDL = low-density lipoprotein

JIA = juvenile idiopathic arthritis

MTX = methotrexate (MTX)

ILAR = International League of Associations for Rheumatology

TC = total cholesterol level

TG = triglycerides

HPLC = High Performance Liquid Chromatography

HDL = high-density lipoprotein

VLDL = very low-density lipoprotein

ROC = receiver operating characteristics

SLE = systemic lupus erythematosus

NSAIDs = nonsteroidal anti-inflammatory drugs

SD = standard deviation

## Competing interests

The author(s) declare that they have no competing interests.

## Authors' contributions

MG, VDA, MOEH were involved in the development of the protocol. MG was responsible to see the patients at outpatient clinic. VDA, EMGS and LCG performed the laboratory tests. MG, CAL and MOEH carried out the literature search. MG, CAL and MOEH prepared and revised the manuscript. CAL coordinated the submission of the article. All authors participated in critical editing of the manuscript and read and approved the final version.

## References

[B1] Rees MM, Rodgers G (1993). Homocysteinemia: Association of metabolic disorder with vascular disease and thrombosis. Thrombosis Research.

[B2] Osganian SK, Stampfer MJ, Spiegelman D, Cutler JA, Feldman HA, Webber LS, Lytle LA, Bausserman L, Nadir PR (1999). Distribution of and factors associated with serum homocysteine levels in children. JAMA.

[B3] Heinecke JW, Rosen H, Suzuki LA, Chait A (1987). The role of sulfur-containing amino acids in superoxide production and modification of low density lipoprotein by arterial smooth muscle cells. J Biol Chem.

[B4] Refsum H, Ueland PM, Nygard O, Volsett SE (1998). Homocysteine and cardiovascular disease. Annu Rev Med.

[B5] Cattaneo M (2000). Hyperhomocysteinemia and atherothrombosis. Ann Med.

[B6] Cassidy JT, Petty RE, Cassidy JP, Petty RE (2001). The Juvenile idiopathic arthritides. Textbook of Pediatric Rheumatology.

[B7] Huemer M, Fodinger M, Huemer C, Sailer-Hock M, Falger J, Rettenbacher A, Bernecker M, Artacker G, Kenzian H, Lang T, Stockler-Ipsiroglu S (2003). Hyperhomocysteinemia in children with juvenile idiopathic arthritis is not Influenced by methotrexate treatment and folic acid supplementation: a pilot study. Clin Exp Rheumatol.

[B8] Huemer M, Huemer C, Ulmer H, Crone J, Fodinger M, Falger J, Sailer-Hock M (2005). No evidence for hyperhomocysteinemia or increased prevalence of genetic polymorphisms in the homocysteine pathway in patients with moderate juvenile idiopathic arthritis. J Rheumatol.

[B9] Petty RE, Southwood TR, Baum J, Bhettay E, Glass DN, Manners P, Maldonado-Cocco J, Suarez-Almazor M, Orozco-Alcala J, Prieur AM (1998). Revision of the proposed classification criteria for juvenile idiopathic arthritis. Durban,1997. J Rheumatol.

[B10] Pfeiffer CM, Huff DL, Gunter EW (1999). Rapid and accurate HPLC assay for plasma total homocysteine and cysteine in a clinical laboratory setting. Clin Chem.

[B11] Del Rincon I, Williams K, Stern MP, Freeman GL, Escalante A (2001). High incidence of cardiovascular events in a rheumatoid arthritis cohort not explained by traditional cardiac risk factors. Arthritis Rheum.

[B12] Nicola PJ, Maradit-Kremers H, Roger VL, Jacobsen SJ, Crowson CS, Ballman KV, Gabriel SE (2005). The risk of congestive heart failure in rheumatoid arthritis: a population-based study over 46 years. Arthritis Rheum.

[B13] Casanueva V, Cid X, Cancino M, Borzone L, Cid L (2003). Serum homocysteine in children and adolescents. Relation with family history of cardiovascular disease. Rev Med Chil.

[B14] Shen MH, Chu NF, Wu DM, Chang JB (2002). Plasma homocysteine, folate and vitamin B12 levels among school children in Taiwan: The Taipei Children Heart Study. Clin Biochem.

[B15] De Laet C, Wautrecht JC, Brasseur D, Dramaix M, Boeynaems JM, Decuyper J, Kahn A (1999). Plasma homocysteine concentration in a Belgian school-age population. Am J Clin Nutr.

[B16] Ray JG, Laskin CA (1999). Folic acid and homocysteine metabolic defects and the risk of placental abruption, pre-eclampsia and spontaneous pregnancy loss: a systematic review. Placenta.

[B17] Volset SE, Refsum H, Irgens LM, Emblem BM, Trerdal A, Gjessing HK, Monsen AL, Ueland PM (2000). Plasma total homocysteine, pregnancy complications, and adverse pregnancy outcomes: the Hordaland Homocysteine study. Am J Clin Nutr.

[B18] Klee GG (2000). Cobalamin and folate evaluation: measurement of methylmalonic acid and homocysteine vs vitamin B12 and folate. Clin Chem.

[B19] Huemer M, Ausserer B, Graninger G, Hubmann M, Huemer C, Schlachter K, Tscharre A, Ulmer H, Simma B (2005). Hyperhomocysteinemia in children treated with antiepileptic drugs is normalized by folic acid supplementation. Epilepsia.

[B20] Afeltra A, Vadacca M, Conti L, Galluzzo S, Mitterhofer AP, Ferri GM, Del Porto F, Caccavo D, Gandolfo GM, Amoroso A (2005). Thrombosis in systemic lupus erythematosus: congenital and acquired risk factors. Arthritis Rheum.

[B21] Hoekstra M, Haagsma CJ, Doelman CJ, Van der Laar MA (2005). Intermittent rises in plasma homocysteine in patients with rheumatoid arthritis treated with higher dose methotrexate. Ann Rheum Dis.

[B22] Van Ede AE, Laan RFJM, Blom HJ, Boers GHJ, Haagsma CJ, Thomas CMG, De Boo TM, van de Putte LB (2002). Homocysteine and folate status in methotrexate-treated patients with rheumatoid arthritis. Rheumatology.

[B23] Fijnheer R, Roest M, Haas FJ, De Groot PG, Derksen RH (1998). Homocysteine, methylenetetrahydrofolate reductase polymorphism, antiphospholipid antibodies and thromboembolic events in systemic lupus erythematosus: a retrospective cohort study. J Rheumatol.

[B24] Chiang PK, Gordon RK, Tal J, Zeng GC, Doctor BP, Pardhasaradhi K, McCann PP (1996). S-Adenosylmethionine and methylation. FASEB J.

[B25] Friso S, Jacques PF, Wilson PW, Rosenberg IH, Selhub J (2001). Low circulating vitamin B6 is associated with elevation of the inflammation marker C-reactive protein independently of plasma homocysteine levels. Circulation.

[B26] Svenson KL, LIthell H, Hallgren R, Selinus I, Vessby P (1987). Serum lipoprotein in active rheumatoid arthritis and other chronic inflammatory arthritides. I. Relativity to inflammatory activity. Arch Intern Med.

[B27] Yoo WH (2004). Dyslipoproteinemia in patients with active rheumatoid arthritis: effects of disease activity, sex, and menopausal status on lipid profile. J Rheumatol.

[B28] Bakkaloglu A, Kirel B, Ozen S, Saatci U, Topaloglu R, Besbas N (1996). Plasma lipids and lipoproteins in juvenile chronic arthritis. Clin Rheumatol.

[B29] Ilowite NT, Samuel P, Ginzler E, Jacobson MS (1988). Dyslipoproteinemia in pediatric systemic lupus erythematosus. Arthritis Rheum.

[B30] Urban M, Pietrewicz E, Gorska A, Glowinska B (2004). Lipids and homocysteine level in juvenile idiopathic arthritis. Pol Merkuriusz Lek.

